# The Use of Antimony

**Published:** 1890-03-01

**Authors:** 


### USE OF ANTIMONY.

Some time ago Dr. K. Spender, of Bath, pointed out in the *Practitioner* that antimony, if frequently repeated in small doses, such as 1-16 part of a grain of tartar emetic every two or three hours, has the power of completely dissipating early local inflammations. In the last report of the "Civil Medical Department of H.H. the Nizam's dominions," we find corroborative evidence of this. Dr. Laurie, the medical officer says, "We have gradually extended its use, and have now come to look upon antimony as one of the most valuable drugs we possess, and as useful in local inflammations not depending on specific or septic causes, as quinine is in malarious fever." It is believed there must be something more than the diaphoretic action of antimony to effect this. It may be given without fear of causing nausea, diarrhœa, or depression, in fact it increases the frequency of the heart's action. It has been found to arrest the mucous-enteritis of children when nothing else will. It has also been found to cut typhoid fever short. Tolerance of the drug is very soon established, and it can be administered if necessary with cardiac tonics. Now there is nothing new in the employment of antimony to arrest inflammations. Dr. Lauder Brunton says in his "Pharmacology, Therapeutics, and Materia Medica," the diaphoretic action of antimony has been used to arrest inflammations such as catarrh and to check feeble conditions. And antimony forms a part of the once famous "James powder." Ringer, in his "Handbook of Therapeutics," says antimony will shorten and moderate attacks of feverish cold, tonsillitis, pleurisy, orchitis, bronchitis, puerperal peritonitis, inflammation of the heart, whitlow, and other inflammatory affections. And Napheys, in "Medical Therapeutics," mentioned the power of antimony in subduing the inflammatory pain of acute pleurisy. There would seem, however, as above stated, to be something more than the mere diaphoretic action, if, as the Residency surgeon of Hyderabad declares, it is so useful in maladies for which *a priori* it would seem to be unsuited. If antimony is useful in the mucus enteritis of children it will be a powerful assistance in this dangerous malady, especially in India. And if antimony has the power of checking typhoid fever it will prove one of the great therapeutical advances of the age. But Dr. Laurie is an acute observer, and anything from him must demand attention.

